# Treatment-seeking for febrile illness in north-east India: an epidemiological study in the malaria endemic zone

**DOI:** 10.1186/1475-2875-8-301

**Published:** 2009-12-17

**Authors:** Himanshu K Chaturvedi, Jagadish Mahanta, Arvind Pandey

**Affiliations:** 1 National Institute of Medical Statistics, ICMR, New Delhi, India; 2 Regional Medical Research Centre, N.E. Region, Dibrugarh, Assam, India

## Abstract

**Background:**

This paper studies the determinants of utilization of health care services, especially for treatment of febrile illness in the malaria endemic area of north-east India.

**Methods:**

An area served by two districts of Upper Assam representing people living in malaria endemic area was selected for household survey. A sample of 1,989 households, in which at least one member of household suffered from febrile illness during last three months and received treatment from health service providers, were selected randomly and interviewed by using the structured questionnaire. The individual characteristics of patients including social indicators, area of residence and distance of health service centers has been used to discriminate or group the patients with respect to their initial and final choice of service providers.

**Results:**

Of 1,989 surveyed households, initial choice of treatment-seeking for febrile illness was self-medication (17.8%), traditional healer *(Vaidya)*(39.2%), government (29.3%) and private (13.7%) health services. Multinomial logistic regression (MLR) analysis exhibits the influence of occupation, area of residence and ethnicity on choice of health service providers. The traditional system of medicine was commonly used by the people living in remote areas compared with towns. As all the febrile cases finally received treatment either from government or private health service providers, the odds (Multivariate Rate Ratio) was almost three-times higher in favour of government services for lower households income people compared to private.

**Conclusion:**

The study indicates the popular use of self-medication and traditional system especially in remote areas, which may be the main cause of delay in diagnosis of malaria. The malaria training given to the paramedical staff to assist the health care delivery needs to be intensified and expanded in north-east India. The people who are economically poor and living in remote areas mainly visit the government health service providers for seeking treatment. So, the improvement of quality health services in government health sector and provision of health education to people would increase the utilization of government health services and thereby improve the health quality of the people.

## Background

Malaria is one of the major public health problems of many countries of world [1-3]. According to WHO report, almost 300 million cases of malaria occur worldwide and more than one million people die due to malaria every year [4]. In India, malaria is reported from many states, but several deaths of epidemic proportion reported every year from different parts of north-east India [5-7]. Assam, being the most populated (27.85 million) and geographically second largest State (78,523 km^2^) of north-east India, is contributing more than 5% of the total number of cases of malaria reported in India. Most districts of the State are malaria-endemic and many pockets in forest, forest fringes, and foothill villages along the inter-country/inter-state border are vulnerable to malaria outbreak as reported [8]. Chloroquine-resistant malaria is widespread in the State, and decreased sensitivity to other anti-malarial drugs has been documented [9]. The malaria cases are detected throughout the year [10], but higher incidence is reported during May to October [11]. There is often delay in reporting illness to health care providers and during these periods certain unorthodox practices are adopted as cost-saving measures.

Although investment in health sectors has been gradually increasing and many health programmes to control malaria, such as spraying DDT in high-risk areas, use of impregnated-bed nets and mass collection of blood slides form the community, have been launched by the government, but there is no appreciable change in malaria incidence. A clear understanding about the health-seeking behaviour of households is crucial for an effective control programme of malaria. For instance, the implementation of current strategy for the control of malaria is mostly based on early diagnosis. It is important to know the factors that determine the utilization of the services of a health care provider for suspected cases of malaria so that an effective strategy can be developed.

Utilization patterns of health service mainly depend on quality of service offered by health care providers and whether it is affordable by the individual user. Moreover, impact of quality differences on the use of health care facilities is to be assessed simultaneously with household and individual characteristics affecting the utilization. The preference of one health care provider over another will be affected not only by facility characteristics (quality of service and cost of service), but also by the individual characteristics such as preferences, affordability, convenient, education level, and household income.

The present study is an attempt to assess the various options of health care patients normally follow for seeking treatment of febrile illness or malaria and also estimate the factors associated with the choice of health services. In the endemic areas, fever is the most common symptom for suspected cases of malaria and is, therefore, being used interchangeably [12].

## Methods

### Study design

Observing high incidence of malaria, two districts of upper Assam, namely Tinsukia and Golaghat, were selected as study area for conducting the survey. A sample of 2,000 households was worked out to capture the variability in health care services and its utilization for both the districts. In the first stage, 50 PSUs (census villages/wards) were selected randomly from each district by using the method of probability proportional to size (PPS) sampling and 20 households from each PSU were selected in a second stage by systematic sampling. The household listing of selected PSUs was prepared during the survey by visiting house-to-house and enquiring whether anybody had malaria or fever in last three months. Those households who reported a fever case and also described the symptom of disease before seeking treatment were recorded in the household listing. A sample of 20 such households, who had received treatment of fever, was selected from the household listing by using systematic sampling. The limitation of such a technique without parasitological analysis is obvious, but in the rural community without laboratory facilities, malaria is diagnosed by the symptoms and clinical manifestations of the disease.

The heads of the selected households, or the most senior person, were interviewed by the trained field workers to collect the general information, whereas specific information about the treatment of fever was collected from the individual patient or mother of sick child in a pre-tested questionnaire. The questionnaire included information about the household, such as household income, education, ethnicity and the use of various options of health care providers for seeking treatment of fever, awareness about malaria, traditional belief and practices, distance of nearest health center (government/private) from residence.

### Health services

Health care delivery in north-east India is mainly provided through the government, private practitioners and private industries. A structural approach to provide health services by population unit is adopted by the government, which is similar to the all-India model (World Bank 1997). The government health care system is mainly categorized in four levels, based upon amenities and service facilities. According to the facility levels, they are district hospitals, community health centers (CHC), primary health centers (PHC) and sub-centers (SHC). The second largest health care service providers are private hospitals of tea gardens, oil and coal industries, including the health clinics of private practitioners.

The traditional system on the other hand comprises the *Vaidya *(herbalists) and priests. The traditional Vaidya mainly use herbs for the preparation indigenous medicine and to provide treatment of diseases. The priests (*ojha*) are believed to 'invoke the spiritual powers' of a deity in diagnosis and treatment of disease. They perform spiritual prayer and also sacrifice of birds or animals during prayer to cure the disease. This system is still being practiced in remote areas and is popular among some tribal communities.

Self-medication is rapidly increasing among all sections of the society, because it saves consultation fees and traveling cost to the health center. In remote areas, the medicines for fever are available and sold freely in the general shop. In towns, the scenario is different, the people describe the symptoms to the drug store operator, who may not necessarily be a pharmacist, and obtain suggestions for drugs to purchase.

### Data analysis

Data collected from the 1989 households in the survey were analysed to identify the possible associated factors with utilization of health services. Statistical tests were applied to examine the association and differences, wherever applicable [13,14]. The geographical area of selected PSUs (villages/wards) was categorized as town, flood, forest, tea garden, and others (Plain area) on the basis of information collected in the survey. Those villages affected by flood every year were categorized as flood area, whereas villages located inside or close to forest were categorized as forest area. Occupation was mainly classified as service (permanent job with regular income and pensioners), self-employed (businessman and professionals), farmer (agriculture), housewives, and others (unemployed, labourers, students). The majority of the households were Hindu (93.6%). Ethnicity of the household respondents was grouped as Assamese (non-tribal), Assam tribes (including Missing, Sonowal, Kachari), Tea tribes of Assam (mainly confined to tea gardens), Bengali (Hindu family), Nepali (originally from Nepal) and others (mostly people who came from other States of India such as Rajasthan, Bihar, Nagaland).

Multinomial logistic regression analysis was used to estimate the rate ratios with respect to indicator/reference category for comparison and identification of actual associates [15]. While seeking treatment of fever, the individual has used more than one option of available treatment. In the model, initial choice of health care service providers, such as traditional system, self-medication, government and private health service, were taken as dependent (or outcome) variable and all other variables viz. residence area, gender, ethnicity, education, occupation, age and income of household, were included as independent variable. However, the analysis was repeated again with the final choice of treatment option (i.e. government or private) used by the individual to know the differences. The Statistical software, SPSS 12.0 was used for statistical analysis.

### Ethical issues

The study was conducted after obtaining approval of the Scientific Advisory Committee (SAC) of the Indian Council of Medical Research. Ethical advice was followed for conducting the survey. The district health administrations and tea garden authorities were also informed to provide local support during the study. At village level, a verbal consent of the village headman was obtained and he was consulted before conducting the survey. Before questioning, the households were apprised in the understandable local language regarding the purpose of visit by the field investigator. The interview was conducted after obtaining consent of the household respondent.

## Results

**Figure 1:**
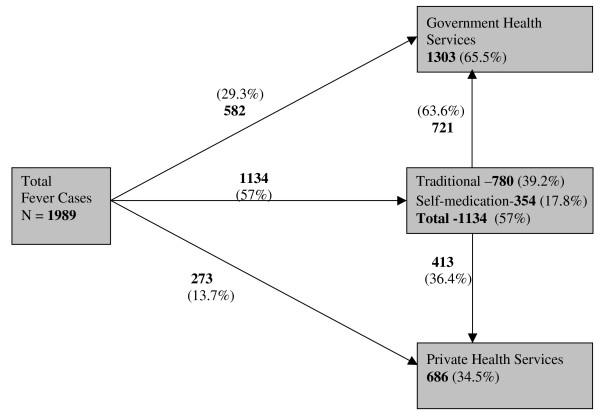
**Diagrammatic presentation of types of health service utilization by the fever cases of households in the malaria endemic areas**.

The general characteristics of household respondents such as age, household income, education, occupation, area of residence, health care services used, distance of nearest health center (government/private) from residence, are presented district-wise for comparison (Table 1). The mean household income was Rs. 2,534 ± 1,816 (INR) per month, but it was significantly lower in Tinsukia district. Variability in monthly household income was high as is reflected by the standard deviation. About 47% of respondents were illiterate and 10% educated up to secondary and above. A majority of the respondents were farmers (38.6%), followed by service class (22%) and self-employed (12.6%) people. Most of the respondents were Hindu (93.6%) and ethically belong to different tribal and non-tribal communities of Assam. The residential area of households was categorized as town (12%), forest (23.0%), flood (22.0%), tea garden (26.3%) and other (16.7%) plain area. The health care services attended initially for seeking treatment of febrile illness were traditional system (39.2%), government health services (29.3%), private health services (13.7%) and self-medication (17.8%) (Figure 1). Except self-medication, the use of health services differs significantly between the tow districts. The average distance to the nearest health center (Government/Private) was 4.2 ± 5.6 km, which was slightly lower for Golaghat district.

**Table 1:** General characteristics of household, socio-economic status, area of living and age and gender distribution of febrile case in the two selected districts.

Variables	Golaghat	Tinsukia	Total
No. of households	995	994	1989

No. of respondents	995	994	1989

Non-response	5	6	11

Gender of respondents			
Female	259	313	572
Male	736	681	1417

Age of respondents (in Years, Mean ± SD)	41.5a ± 11.6	40.8a ± 12.5	41.1 ± 12.0

Household Income (Rs./month) Mean ± SD	2776f ± 1808	2293f ± 1793	2534 ± 1816

Education of respondent	Percentage of respondents (N)		
Illiterate	41.5 (413)	52.5 (522)	47.0 (935)
Primary	22.3 (222)	15.5 (154)	18.9 (376)
Middle	24.5 (244)	23.7 (236)	24.1 (480)
Secondary+	11.7 (116)	8.3 (82)	10.0 (198)

Occupation of respondent			
Service	15.5 (154)	28.5 (283)	22.0 (437)
Self-employed	14.0 (139)	11.2 (111)	12.6 (250)
Farmer	42.0 (418)	35.2 (350)	38.6 (768)
Housewife	23.1 (230)	19.3 (192)	21.1 (422)
Others	5.4 (54)	5.8 (58)	5.6 (112)

Religion			
Hindu	91.3 (908)	95.9 (953)	93.6 (1861)
Christian	0.2 (2)	2.1 (21)	1.2 (23)
Muslim	8.5 (85)	2.0 (20)	5.3 (105)

Area of residence			
Town	5.9 (59)	18.1 (180)	12.0 (239)
Forest	26.4 (263)	19.6 (195)	23.0 (458)
Flood	24.0 (239)	19.9 (198)	22.0 (437)
Tea garden	20.1 (200)	32.4 (322)	26.3 (522)
Others	23.5 (234)	10.0 (99)	16.7 (333)

Health Care Service	Percentage of user (N)		
Traditional *Vaidya*	35.3 (351)	43.2 (429)	39.2 (780)
Government	36.3 (361)	22.2 (221)	29.3 (582)
Private	9.9 (99)	17.5 (174)	13.7 (273)
Self-medication	18.5 (184)	17.1 (170)	17.8 (354)

Gender of patient (fever case)			
Male	58.1 (578)	59.7 (593)	58.9 (1171)
Female	41.9 (417)	40.3 (401)	41.1 (818)

Age of Patients (Years)	23.0a ± 18.3	23.1a ± 19.1	23.1 ± 18.7

Distance of Health Center (km) (Mean ± SD)	3.5f ± 2.8	4.9f ± 6.4	4.2 ± 5.6

Values marked by similar letter (a, b, or c; not in superscript) shows no significant difference; e: P < 0.05; f: P < 0.01;

Health care services used by the households and its differences across socio-demographic characteristics of households, residential area and patient's characteristics are presented with statistical analysis (Table 2). The association of health care services was significant with all the variables, except gender of patients. The traditional system was the first choice of treatment among majority of people residence in tea garden (60%), flood (43.7%) and forest (33.8%) areas. However, self-medication was popular in forest (25.8%) and town (25.5%) areas. A decreasing pattern in the use of the traditional system was observed with increasing level of education of household heads. It was also recorded to be high among farmers (42.5%), tea tribes (57.1%) and Nepali (50.5%) communities. As an initial choice, self-medication was high among higher educational groups, i.e. secondary+ (20.7%); farmers (24.3%); Nepali (26.9%) and Assamese (20.2%) communities. It was also recorded high among higher household income groups (21.4%). The increasing pattern of self-medication was observed with increase age group of patients.

**Table 2:** Health care services utilization for seeking treatment (P) and its association with residential areas of living and socio-demographic characteristics of household head and patients.

Variables	N	Health Care Services					
		
		Initial Choice				Final Choice	
		
		Traditional*	Govt. Service^$^	Private Service^#^	Self-medication^@^	Govt. Service^$^	Private Service^#^
		P (%)	P (%)	P (%)	P (%)	P (%)	P (%)
**Location**			Residential	Area			
Golaghat	995	35.3	36.3	9.9	18.5a	78.5	21.5
Tinsukia	994	43.2	22.2	17.5	17.1a	52.5	47.5

χ^2^--test		62.6f				148.5f	

**Area of living**							
Town	239	10.5	34.7a	29.3	25.5a	49.0	51.0
Forest	458	33.8	31.7a	8.7a	25.8a	80.6a	19.4a
Flood	437	43.7	31.8a	7.1a	17.4b	78.9a	21.1a
Tea garden	522	60.0	14.2	17.8	8.0	43.1	56.9
Others	333	28.8	42.3	11.7	17.1b	74.2	25.8

χ^2^--test		311.1f				236.9f	

**Education of head**		Information of Household Head					
Illiterate	935	41.3ab	26.1	13.7a	18.9ab	66.5a	33.5a
Primary	376	43.9a	31.1a	9.8	15.2c	66.2ab	33.8ab
Middle	480	38.5b	29.8a	15.2ab	16.5ac	64.6ab	35.4ab
Secondary+	198	22.2	39.4	17.7b	20.7b	61.6b	38.4b

χ^2^--test		225.2f				2.02d	

**Occupation**							
Service	437	44.4a	21.1	24.5	10.1	34.3	65.7
Self-employed	250	21.6	46.8	15.6a	16.0a	68.0a	32.0a
Farmer	774	42.5a	27.6a	5.6b	24.3b	84.9	15.1
Housewife	422	38.2b	29.1a	18.0a	14.7a	58.8	41.2
Others	106	39.6ab	34.0a	7.5b	18.9ab	73.6a	26.4a

χ^2^--test		182.4f				328.9f	

**Ethnic group**							
Assamese (Non-tribe)	445	36.0a	33.7a	10.1	20.2a	70.1a	29.9a
Assam tribes	353	40.2a	33.4a	6.8a	19.5a	85.6b	14.4b
Tea tribes of Assam	487	57.1	14.8b	20.3b	7.8	36.3	63.7
Nepali	212	50.5	17.9b	4.7a	26.9	83.0b	17.0b
Others	492	18.9	41.5	19.3b	20.3a	68.3a	31.7a

χ^2^--test		270.7f				280.7f	

**Household Income **			(INR)				
≤ 2000	1113	45.2	26.7a	13.3a	14.8	65.0	35.0
2001--4000	628	31.8a	33.6b	12.9a	21.7a	71.0	29.0
4001 +	248	31.0a	29.8ab	17.7	21.4a	54.0	46.0

χ^2^--test		45.6f				23.05f	

**Gender of Patients**		Patients	Information				
Male	1171	40.1a	29.5a	12.6a	17.8a	66.7a	33.3a
Female	818	37.9a	29.0a	15.4a	17.7a	63.8a	36.2a

χ^2^--test		3.51d				1.76d	

**Age of patients**							
≤ 10(in Years)	713	39.0	29.9a	16.5	14.6	61.7	38.3
11 -- 30	658	42.7	29.6a	11.9a	18.5a	66.6a	33.4a
31+	618	35.8	31.1a	12.5a	20.7a	68.8a	31.2a

χ^2^--test		19.8f				7.79e	

**Distance of Health Center**							
< 5 Km.	1391	38.0a	28.5a	16.5	17.0a	59.2	40.8
≥ 5 Km.	598	42.0a	31.1a	7.4	19.6a	80.3	19.7

χ^2^--test		29.4f				82.4f	

N: Number of respondents; *Traditional healer/*Vaidya; *$ PHC/CHC/Sub Center/District Hospital; # Private Dispensary/Hospital; @ Medicines without consulting a doctor; CI: 95% Confidence Interval; Values marked by similar letter (a, b, or c; not in superscript) shows no significant difference; d: P > 0.05; e: P < 0.05; f: P < 0.01

Government health service, as initial choice of seeking treatment, was high in all the residential areas (32% to 42%), except in tea garden areas (14.2%). It was also shown to be low among tea tribes of Assam (14.8%). Similarly, the private health services were commonly used in town (29.3%) and tea gardens (17.8%). Utilization of private health services was also high among tea tribes (20.3%) and higher household income group (17.7%).

Analysis of final choice of seeking treatment indicated the significant association with location, area of residence, occupation, ethnicity, household income and distance of nearest health center. However, the association of education and gender of patients was not significant. The utilization of government health services was significantly higher in Golaghat district (78.5%) compared to Tinsukia (52.5%). It was also recorded high in forest (80.6%) and flood area (78.9%). The private health services were used commonly in tea garden areas, especially by the tea tribes (Table 2).

The results of multinomial logistics regression (MLR) analysis with initial and final treatment-seeking choice are presented in Table 3. The estimated coefficients (β) of the variables in the model indicate how changes in each of the variable affect the household choice of health service providers for seeking treatment of febrile illness. Except for gender of patients, a significant effect of location, residential area, education, occupation, ethnicity, age of patients and household income was recorded with the dependent variables, i.e. health care services. Multivariate rate ratios (MRR) for traditional system as initial choice was significantly higher for residential area: tea garden (5.6), flood (3.2), forest (1.5); level of education: literate (1.7); ethnic group: Assamese (1.5) and tea tribes (3.4); and distance of health center: ≥ 5 km (1.4) compare with respective reference. It clearly indicates that the use of traditional system of medicine was much higher in tea garden, forest and flood affected area with compare to town and others. The MRR of government service as initial choice was significantly higher for education: literate (1.4); occupation: service and self-employed (2.4); others (1.9); and low household income (1.8). The household income was one of the important determinants for utilization of government services. However, there was no significant effect of residential area and ethnic background of people. The private services as initial choice shows the significantly higher value of MRR (odd) with location: Golaghat (1.5); education: literate (1.4); occupation: service and self-employed (4.1) and others (3.4); ethnic group: tea tribes (2.6); and age of patients: lower age group (1.9) compare to respective reference. However, there was no significant effect of residential area, household income and gender of patients on utilization of private health services.

**Table 3:** Multinomial logistic regression analysis to estimate the β coefficient and multivariate rate ratios (MRR) for seeking treatment of febrile illness.

Variables	Health Care Services							
	
	Initial Choice (Ref. Self-medication*)						Final Choice (Ref.: Private Service#)	
	
	Traditional *Vaidya*		Govt. Service^$^		Private Service^#^		Govt. Service^$^	
	**Est.(β)**	**MRR (95%CI)**	**Est.(β)**	**MRR(95%CI)**	**Est.(β)**	**MRR(95%CI)**	**Est.(β)**	**MRR (95%CI)**

**Location**				**Residential**	**Area**			
Golaghat^a^	0	1.0	0	1.0	0	1.0	0	1.0
Tinsukia	0.14d	1.2 (0.9-1.5)	-0.46f	0.6 (0.5-0.8)	0.38e	1.5 (1.02-2.1)	-1.16f	0.3 (0.25-0.4)

**Area of living**								
Town & others^a^	0	1.0	0	1.0	0	1.0	0	1.0
Forest	0.38e	1.5 (1.01-2.1)	-0.23d	0.8 (0.6-1.14)	-0.41d	0.7 (0.4-1.1)	0.32d	1.4 (0.99-1.9)
Flood	1.17f	3.2 (2.2-4.8)	0.26d	1.3 (0.9-1.9)	0.02d	1.0 (0.6-1.8)	-0.13d	0.9 (0.6-1.2)
Tea garden	1.72f	5.6 (3.4-9.1)	-0.15d	0.9 (0.5-1.4)	0.33d	1.4 (0.8-2.5)	-0.23d	0.8 (0.6-1.1)

**Education of head**								
Illiterate^a^	0	1.0	0	1.0	0	1.0	0	1.0
Literate	0.54f	1.7 (1.3-2.3)	0.32e	1.4 (1.03-1.9)	0.36e	1.4 (1.0-2.1)	-0.47f	0.6 (0.5-0.8)

**Occupation**								
Farmer^a^	0	1.0	0	1.0	0	1.0	0	1.0
Service & Self-empl.	0.12d	1.1 (0.8-1.6)	0.86f	2.4 (1.6-3.4)	1.41f	4.1 (2.5-6.7)	-1.15f	0.32 (0.2-0.4)
Others	0.61f	1.8 (1.3-2.6)	0.64f	1.9 (1.3-2.7)	1.22f	3.4 (2.1-5.6)	-0.93f	0.4 (0.3-0.5)

**Ethnic group**								
Assamese	0.41f	1.5 (1.1-2.1)	0.09d	1.1 (0.8-1.5)	-0.12d	0.9 (0.6-1.3)	-0.17d	0.8 (0.6-1.1)
Tea tribes of Assam	1.22f	3.4 (2.1-5.5)	0.13d	1.1 (0.7-1.9)	0.94f	2.6 (1.4-4.7)	-1.59f	0.2 (0.14-0.3)
Others^a^(outsider)	0	1.0	0	1.0	0	1.0	0	1.0

**Household Income**								
≤ 2000(INR)	0.28d	1.3 (0.9-2.0)	0.61f	1.8 (1.2-2.8)	0.02d	1.0 (0.6-1.7)	1.08f	2.9 (2.1-4.2)
2001--4000	-0.03d	1.0 (0.6-1.5)	0.32d	1.4 (0.9-2.1)	-0.10d	0.9 (0.5-1.5)	0.69f	2.0 (1.4-2.8)
4001 +^a^	0	1.0	0	1.0	0	1.0	0	1.0

**Gender of Patients**								
Male	0.10d	1.1 (0.8-1.5)	0.04d	1.1 (0.8-1.4)	-0.13d	0.9 (0.6-1.2)	0.07d	1.1 (0.9-1.3)
Female^a^	0	1.0	0	1.0	0	1.0	0	1.0

**Age of Patients**								
≤ 10(in Years)	0.43f	1.5 (1.1-2.2)	0.27d	1.3 (0.9-1.8)	0.62f	1.9 (1.2-2.8)	-0.32d	0.7 (0.6-1.0)
11 -- 30	0.38e	1.5 (1.1-2.0)	-0.04d	1.0 (0.7-1.3)	0.16d	1.2 (0.8-1.8)	-0.22d	0.8 (0.6-1.1)
31+ ^a^		1.0		1.0		1.0		1.0

**Distance of Health Center**								
< 5 Km^a^.	0	1.0	0	1.0	0	1.0	0	1.0
≥ 5 Km.	0.35e	1.4 (1.01 --1.9)	0.16d	1.2 (0.9-1.6)	-0.24d	0.8 (0.5-1.2)	0.48f	1.6 (1.2-2.1)

β:Regression Coefficient; MRR: Multivariate Rate Ratios; CI: 95% Confidence Interval; d: P > 0.05 (not significant); e: P < 0.05; f: P < 0.01; ^a^Reference category.

As a final choice of seeking treatment, the MLR analysis shows the significant effect of location, education, occupation, ethnicity, household income and distance of nearest health center with government health services utilization with reference to private services. The estimated values of β and MRR shows the significant negative or decreasing effect with location: Tinsukia (-1.16; 0.3); education: literate (-0.47 & 0.6); occupation: service & self-employed (-1.15 & 0.32) and others (-0.93 & 0.2); and ethnic group: tea tribes (-1.59 & 0.2) with their respective reference. However, it was almost three times higher in favour of government health services for lower income (≤ 2000 INR) group (2.9). If the distance of health center increases (≥ 5 km), the MRR was significantly higher (1.6) in favour of government health services. The area of residence was not showing any effect with the final choice of two health services i.e. government or private.

## Discussion

A better understanding of health-seeking behaviour of people, especially when suffering from fever, a common symptom of malaria, is important for effective management and control of malaria. This is related with early treatment, accessibility and proper use of drugs. The most common treatment options reported by the households for seeking treatment of fever was use of traditional drugs, self-medication, government and private health services, which was also reported by others in many studies [16-19].

The use of traditional medicines was prevalent in remote areas especially in tea gardens, forest and flood affected areas. It seems that internal factors of the specific geographical region like availability and accessibility of health services; traditional belief and practices was motivating the people for seeking medical assistance from traditional system (Vaidya). Although most of the people received treatment finally from government or private health services, but majority of them initially visited to traditional Vaidya for seeking treatment of febrile illness. The strong belief in traditional system was reflected even among literate people. A study in Tanzania also reported that traditional healers are an important factor of delay for malaria treatment [18].

Self-medication and consultations at medical stores was a common practice in town and forest areas. Such practices created a situation where people wait till the patient become serious enough before reporting to health centers. Obviously, some of these patients develop complications and even die by the time they reach a hospital. No doubt, the 'wait-and-watch' attitude is not only harmful to the patients, but also contributing to increased malaria. These practices are very common not only among un-educated and poor people, but across most of the variables included in the analysis. This was also reported and discussed by others [20-22].

Over all, response of availing government health services was high by the people residing in forest and flood affected area. Moreover, the people from town and tea garden areas were seeking treatment mainly from private health services, because private health services were mainly confined in the town and private tea gardens located outside the town nearby the tea gardens. Despite longer distances and poor connectivity to motorable roads, the accessibility to government health services was recorded high among the people living in forest and flood areas. The increasing level of education of household head has decreasing effect with accessibility of government services. The MRR was also significantly low for literates with reference to illiterates. Household income is one of the important determinants for health service utilization. Similarly, increasing level of household income has decreasing effect with accessibility of government services. It was possibly due to the expensive treatment in private health centers. The socio-economic impact on choice of health services was also discussed by others [23-26].

Early diagnosis of febrile illness or malaria can yield rich dividends for effective management and control of malaria [18,27-29]. Though the distance to a health center is one of the important factors of delay, its effect may be confounded with the place of residence. Residential areas such as forest, flood and tea garden, show the highest preference for traditional system and self-medication, which could be the reason of delay in proper diagnosis. Interestingly, there is no gender bias in the choice of seeking treatment, which is often reported as an important indicator to address this issue [29,30]. However, the decision-making power on treatment-seeking behaviour mainly depends on male family elders [30].

Although treatment expenditure is one of the important components, which influence the household choice of health service providers [21], information could not be collected in this study due to recall bias and lack of records during survey. Moreover, household income is recorded as one of the important determinants influencing the utilization of health services. The identification of various other indicators in this study provides useful information to understand the interlinked behavioural practices related to malaria. As early diagnosis is the main theme of prevention and control of malaria, the effective strategies to provide health services at nearest periphery of village/community and parallel health awareness programme with people participation are needed to circumvent the situation.

## Competing interests

The authors declare that they have no competing interests.

## Authors' contributions

HKC contributed to the conception, preparation of project proposal and design of the study, developed the questionnaire and collected data, and contributed to the analysis, interpretation of the data and to the writing of the paper. JM contributed to the conception and preparation plan of study, developed the questionnaire and the data collection. AP contributed to the interpretation of data and to write the paper. All authors read and approved the final manuscript.
